# Prognostic evaluation of platelet to lymphocyte ratio in patients with colorectal cancer

**DOI:** 10.18632/oncotarget.21141

**Published:** 2017-09-21

**Authors:** Chong Lu, Peng Gao, Yuchong Yang, Xiaowan Chen, Longyi Wang, Dehao Yu, Yongxi Song, Qingzhou Xu, Zhenning Wang

**Affiliations:** ^1^ Department of Surgical Oncology and General Surgery, The First Hospital of China Medical University, Shenyang 110001, PR China

**Keywords:** colorectal cancer, meta-analysis, platelet to lymphocyte ratio, prognosis, TNM staging

## Abstract

Growing evidence indicates that inflammation plays an important role in cancer progression and prognosis; however, the prognostic role of platelet to lymphocyte ratio (PLR) in colorectal cancer (CRC) is unknown. A cohort of 1845 CRC patients from the Department of Surgical Oncology at The First Hospital of China Medical University (CMU-SO) was retrospectively analyzed. Harrell’s concordance index (c-index) was used to determine the optimal cut-off value of PLR and evaluate its predictive ability. Our results from CMU-SO indicated that the overall survival (OS) rate was significantly lower in the high-PLR group compared with the low-PLR group (*P* = 0.001). A similar result was observed for the cancer-specific survival (CSS) rate between these two groups (*P* = 0.001). The multivariate analysis indicated that high PLR was an independent prognostic indicator of poor OS (hazard ratio [HR] = 1.356, 95% confidence interval [CI] = 1.117–1.647, *P* = 0.002) and CSS (HR = 1.364, 95% CI = 1.111–1.675, *P* = 0.003). In addition, the c-indexes of TNM staging combined with PLR were greater than those of TNM staging alone (OS: 0.768 vs. 0.732; CSS: 0.785 vs. 0.746). In conclusion, elevated PLR is a negative prognostic indicator of CRC and may serve as an additional index of the current TNM staging system for predicting CRC.

## INTRODUCTION

Colorectal cancer (CRC) is one of the most common digestive cancers and the third leading cause of cancer-related death in the United States [1]. To date, the TNM staging system has been regarded as the gold-standard method for predicting prognosis and guiding treatment. However, patients with the same TNM stage may have a different clinical prognosis. Therefore, novel biomarkers are required to complement the current TNM staging system and accurately predict the prognosis of CRC.

A growing body of evidence indicates that the host inflammatory response can affect the progression and prognosis of cancer [2, 3]. In fact, previous studies have indicated that inflammatory biomarkers, including neutrophil to lymphocyte ratio [4–6], prognostic nutritional index [7, 8], lymphocyte to monocyte ratio [9, 10] and C-reactive protein [11, 12], can be used in the prognosis of gastrointestinal cancers. However, a consensus on the prognostic value of the platelet to lymphocyte ratio (PLR) in CRC was not reached. Ozawa et al.[13] and Kwon et al.[14] reported that PLR was significantly associated with prognosis in CRC patients, whereas other authors indicated that elevated PLR did not predict poor prognosis [15, 16]. In addition, no studies have evaluated the potential use of PLR as an additional tool in the current tumor staging system, and the optimal cut-off value of PLR for predicting prognosis in CRC remains unknown.

In this study, we retrospectively analyzed a cohort of patients from our institution and explored the optimal cut-off value of PLR for predicting prognosis in CRC. Moreover, we evaluated the additional prognostic value of PLR in the current TNM staging system.

## RESULTS

### Optimal cut-off value of PLR

To date, the optimal cut-off value of PLR for predicting prognosis in CRC remains unknown. We used the Harrell’s C-index (c-index) method to determine the optimal cut-off values of PLR for overall survival (OS) and cancer-specific survival (CSS). We calculated the c-index values for different cut-off values. Our results indicated that the c-indexes for OS and CSS were maximum when PLR was 130 (Supplementary Table 1). Among the 1845 patients from CMU-SO, 1018 patients (55.18%) had a PLR < 130 and 827 patients (44.82%) had a PLR ≥ 130 and were classified into the low-PLR group and high-PLR group, respectively.

### PLR and clinicopathological characteristics

Among 1845 CRC patients from CMU-SO, female patients, larger tumor size, tumor location in the colon, poorer differentiation, deeper depth of tumor, and advanced TNM stages were more frequently observed in the high-PLR group than in the low-PLR group (*P* < 0.001). However, there was no significant difference in age (*P* = 0.721), lymph node metastasis status (*P* = 0.140), and distant metastasis status (*P* = 0.932) between these two groups (Table 1).

**Table 1:** Associations between clinicopathological features and PLR in CRC patients from CMU-SO

Variable	Number (%)	PLR status		*P* value
		Low PLR (%)	High PLR (%)	
Sample size	1845 (100)	1018 (55.2)	827 (44.8)	
Age(y)				0.721
<60	817 (44.3)	447 (43.9)	370 (44.7)	
≥60	1028 (55.7)	571 (56.1)	457 (55.3)	
Gender				<0.001
Male	1044 (56.6)	617 (60.6)	427 (51.6)	
Female	801 (43.4)	401 (39.4)	400 (48.4)	
Tumor size (cm)				<0.001
<4.7	920 (49.9)	584 (57.4)	336 (40.6)	
≥4.7	925 (50.1)	434 (42.6)	491 (59.4)	
Tumor location				<0.001
Colon	775 (42.0)	342 (33.6)	433 (52.4)	
Rectum	1070 (58.0)	676 (66.4)	394 (47.6)	
Differentiation				<0.001
Well - moderate	1684 (91.3)	952 (93.5)	732 (88.5)	
Poor - undifferentiated	161 (8.7)	66 (6.5)	95 (11.5)	
Depth of tumor				<0.001
T1	48 (2.6)	29 (2.8)	19 (2.3)	
T2	346 (18.8)	237 (23.3)	109 (13.2)	
T3	742 (40.2)	389 (38.2)	353 (42.7)	
T4	709 (38.4)	363 (35.7)	346 (41.8)	
Lymph node metastasis				0.140
N0	1079 (58.5)	607 (59.6)	472 (57.1)	
N1	559 (30.3)	310 (30.5)	249 (30.1)	
N2	207 (11.2)	101 (9.9)	106 (12.8)	
Distant metastasis				0.932
Negative	1802 (97.7)	994 (97.6)	808 (97.7)	
Positive	43 (2.3)	24 (2.4)	19 (2.3)	
TNM stage				<0.001
I	311 (16.9)	208 (20.4)	103 (12.5)	
II	758 (41.1)	396 (38.9)	362 (43.8)	
III	733 (39.7)	390 (38.3)	343 (41.5)	
IV	43 (2.3)	24 (2.4)	19 (2.3)	

CMU-SO: Department of Surgical Oncology at The First Hospital of China Medical University; PLR: platelet to lymphocyte ratio.

### PLR and prognosis

**Figure 1:**
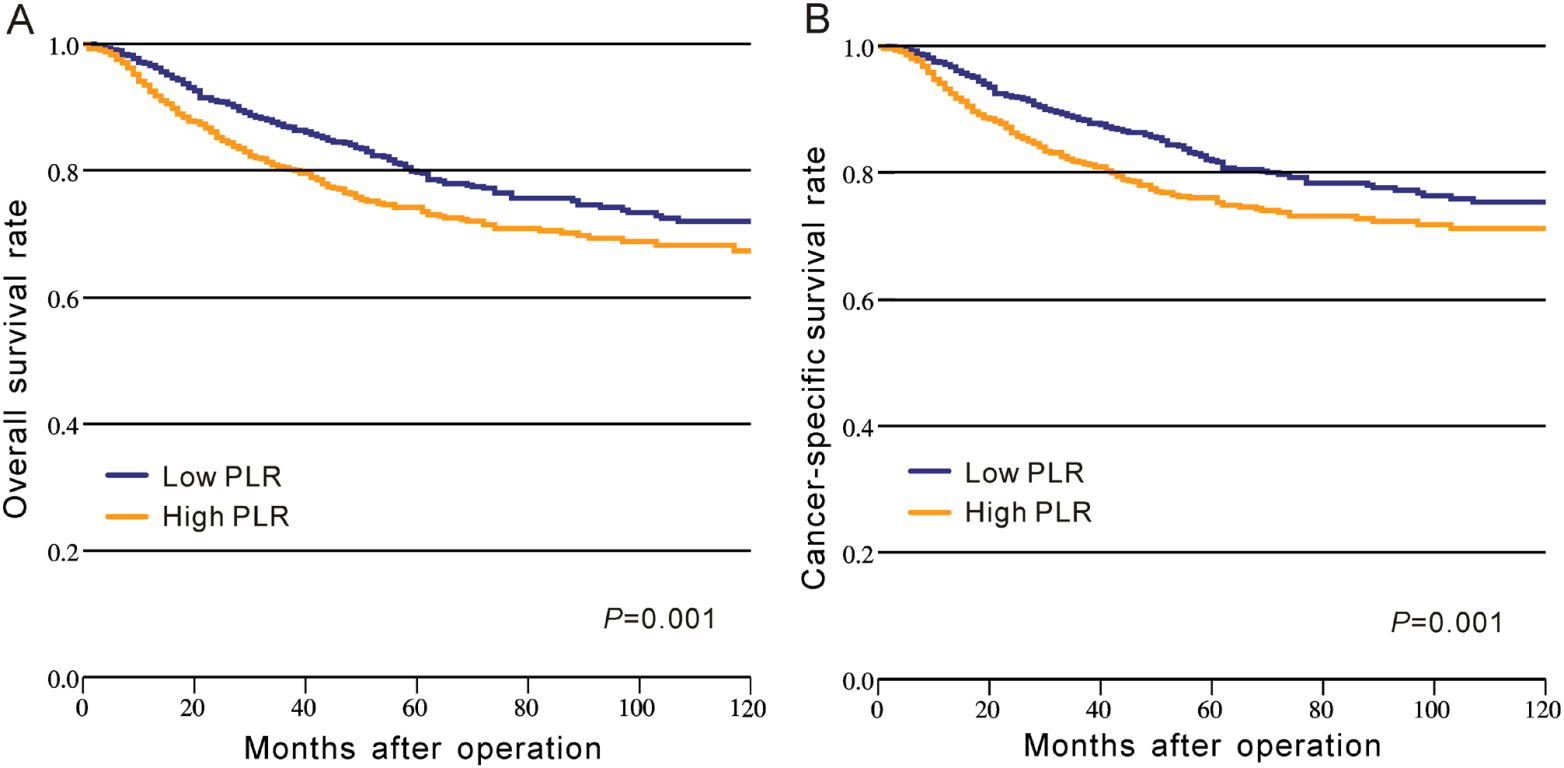
Kaplan–Meier curves of survival based on the platelet to lymphocyte ratio in CRC patients from CMU-SO **(A)** Overall survival; **(B)** cancer-specific survival.

**Figure 2:**
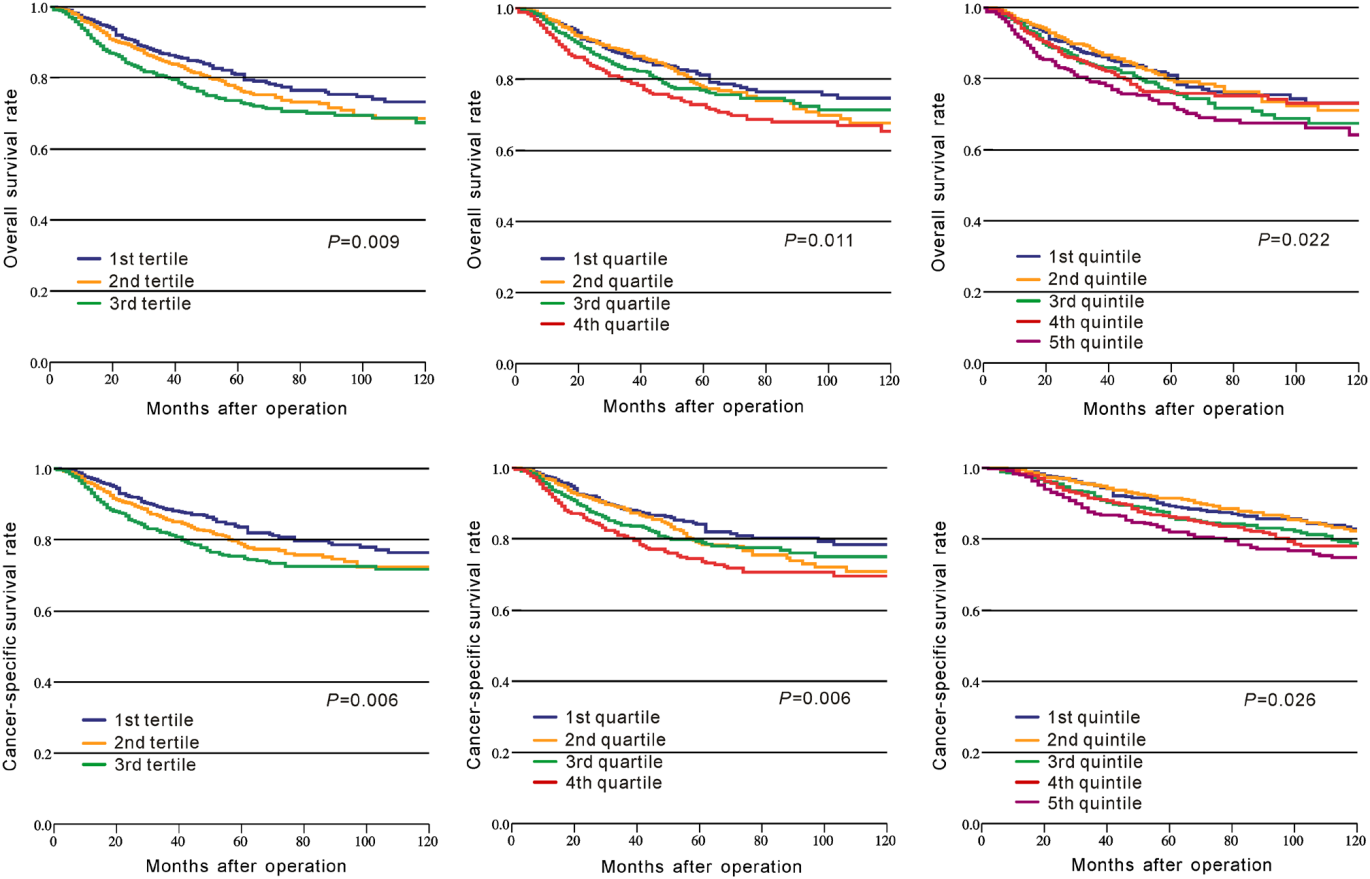
Kaplan–Meier curves of survival based on the platelet to lymphocyte ratio divided in tertiles, quartiles, and quintiles

In CRC patients of CMU-SO, OS rate was significantly lower in the high-PLR group than that in the low-PLR group (*P* = 0.001, Figure 1A, Table 2). A similar result was observed for the CSS rate between these two groups (P = 0.001, Figure 1B, Table 2). Moreover, we divided PLR in tertiles, quartiles, and quintiles in the prediction of OS and CSS, and the results indicated that no matter how to group, PLR was significantly associated with OS and CSS in CRC (P < 0.05, Figure 2).

**Table 2:** Univariate and multivariate survival analyses of OS and CSS in patients with colorectal cancer from CMU-SO

Variable	Overall survival				Cancer-specific survival			
	Univariate		Multivariate		Univariate		Multivariate	
	HR (95% CI)	*P*	HR (95% CI)	*P*	HR (95% CI)	*P*	HR (95% CI)	*P*
Gender		0.018		0.003		0.109		
Female	1		1		1			
Male	1.270 (1.043-1.546)		1.358 (1.113-1.657)		1.185 (0.963-1.459)			
Age (y)		0.144				0.606		
<60	1				1			
≥60	1.156 (0.952-1.405)				1.055 (0.860-1.296)			
Tumor size (cm)		0.717				0.767		
<4.7	1				1			
≥4.7	1.036 (0.855-1.255)				1.031 (0.842-1.264)			
Tumor location		0.319				0.287		
Colon	1				1			
Rectum	1.105 (0.908-1.344)				1.120 (0.909-1.379)			
Differentiation		<0.001		0.001		<0.001		0.004
Well - moderate	1		1		1		1	
Poor - undifferentiated	2.374 (1.816-3.104)		1.569 (1.195-2.060)		2.379 (1.791-3.162)		1.523 (1.141-2.032)	
Depth of tumor		<0.001		0.006		<0.001		0.002
T1	1		1		1		1	
T2	3.150 (0.764-12.984)		2.733 (0.663-11.273)		2.519 (0.606-10.462)		2.143 (0.516-8.909)	
T3	6.173 (1.532-24.868)		3.162 (0.783-12.772)		5.363 (1.330-21.629)		2.677 (0.661-10.835)	
T4	9.321 (2.312-37.569)		4.180 (1.033-16.911)		8.541 (2.118-34.446)		3.712 (0.916-15.044)	
Lymph node metastasis		<0.001		<0.001		<0.001		<0.001
N0	1		1		1		1	
N1	4.682 (3.668-5.976)		4.229 (3.300-5.421)		5.434 (4.150-7.116)		4.859 (3.695-6.390)	
N2	11.926 (9.122-15.592)		10.386 (7.870-13.708)		14.189 (10.597-19.000)		11.834 (8.760-15.986)	
Distant metastasis		<0.001		<0.001		<0.001		<0.001
Negative	1		1		1		1	
Positive	4.046 (2.628-6.230)		2.222 (1.437-3.436)		4.316 (2.772-6.718)		2.310 (1.476-3.615)	
TNM stage		<0.001				<0.001		
I	1				1			
II	1.752 (1.058-2.901)				2.241 (1.206-4.165)			
III	9.180 (5.772-14.599)				13.260 (7.437-23.641)			
IV	17.507 (9.451-32.430)				26.045 (12.783-53.064)			
PLR		0.001		0.002		0.001		0.003
<130	1		1		1		1	
≥130	1.364 (1.127-1.653)		1.356 (1.117-1.647)		1.409 (1.150-1.727)		1.364 (1.111-1.675)	

CI: confidence interval; CMU-SO: Department of Surgical Oncology at The First Hospital of China Medical University; HR: hazard ratio; PLR: platelet to lymphocyte ratio.

In addition, the results of Cox multivariate analysis indicated that elevated PLR was an independent prognostic factor for poor OS (HR = 1.356, 95% CI = 1.117–1.647, P = 0.002) and CSS (HR = 1.364, 95% CI = 1.111–1.675, *P* = 0.003, Table 2).

### Prognostic ability of TNM staging combined with PLR (TNM+PLR)

**Figure 3:**
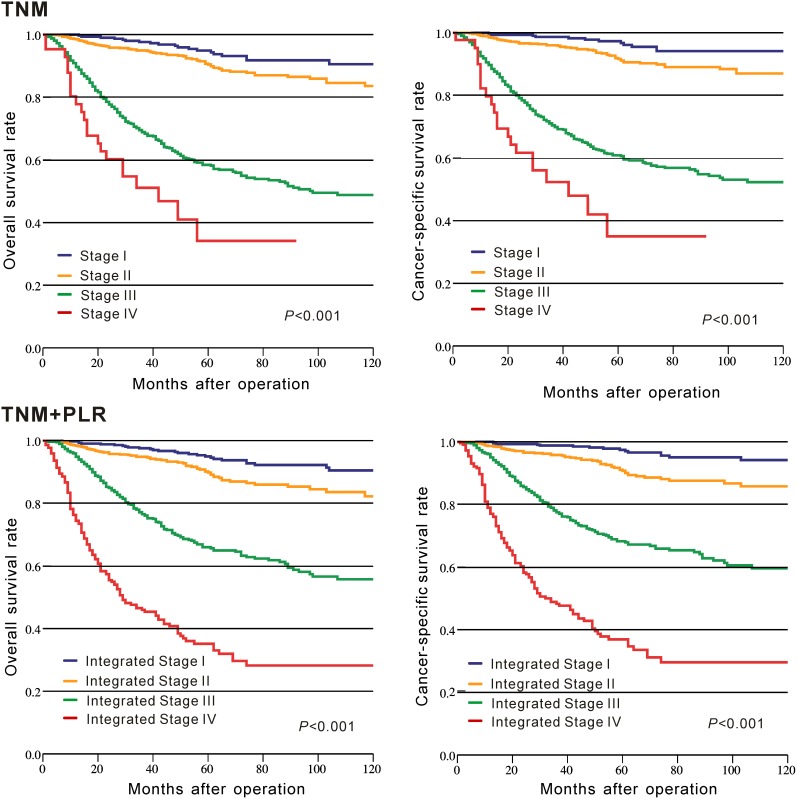
Kaplan–Meier curves of survival based on TNM staging and TNM staging combined with the platelet to lymphocyte ratio in CRC patients from CMU-SO

The c-indexes of TNM+PLR and TNM staging alone for OS and CSS were used to assess and compare their prognostic capacity. Our result indicated that the c-indexes were greater in TNM+PLR (OS: 0.768 vs. 0.732; CSS: 0.785 vs. 0.746) than those in TNM staging alone. Moreover, the K-M curve indicated that TNM+PLR staging had a better discrimination to divide patients with different prognosis into different groups compared with TNM staging alone (Figure 3).

## DISCUSSION

In this study, we retrospectively analyzed a cohort of patients from our institution. We found that elevated PLR was an independent prognostic factor for poor OS and CSS. Furthermore, elevated PLR was significantly associated with advanced tumor features, including larger tumor size, poorer differentiation, deeper depth of tumor, and advanced TNM stages. These results demonstrated that elevated PLR was a predictive indicator of poor prognosis and was significantly associated with advanced tumor features in patients with CRC.

The mechanisms underlying the relationship between elevated PLR and poor prognosis of CRC are still unknown. Recent studies have demonstrated that platelets can prevent the death of tumor cells by natural killer cells and platelets secrete angiogenic and tumor growth factors, including vascular endothelial growth factor and platelet-derived growth factor, and thus promote the growth, progression, and spread of tumor [17–20]. Furthermore, it has been reported that thrombocytosis is associated with poor prognosis in CRC [21, 22]. Lymphocytes are the main components of the immune system of the host and can eliminate cancer cells and prevent tumor progression [23]. Some studies have shown that low lymphocyte count was a poor prognostic factor in patients with CRC [24, 25]. Therefore, elevated PLR, relatively high platelet counts, and low lymphocyte counts may predict poor prognosis of CRC. On the other hand, our results confirmed that elevated PLR was significantly associated with advanced tumor features, including poorer differentiation, deeper depth of tumor, and advanced TNM stages. Therefore, elevated PLR was associated with the extent of tumor progression and consequently may affect the survival of patients with CRC. However, it is of note that malignant tumor usually induces a hypercoagulable state in the host [26] and results in thrombocytosis. Whether elevated PLR is a cause or consequence of cancer progression is unknown and needs to be elucidated.

Controversy still exists on the optimal methods that should be used to calculate the cut-off value and the optimal cut-off value of PLR for predicting prognosis in patients with CRC. Choi et al.[27] used the method of maximizing log-rank test statistics, some authors used receiver operating characteristic curve analysis [28, 29], whereas Li et al.[16] used X-tile software to determine the cut-off value. To date, there is no uniform approach to determining a universal cut-off value suitable for all patient cohorts. In our study, c-index, which is a measure of discrimination and a commonly used method to evaluate the predictive ability of models, was used to find the optimal cut-off value among the 1845 patients with CRC. However, whether the cut-off value identified in the patient cohort from our institution can be applied to other independent cohorts needs to be further investigated.

Some pooled studies explored the prognostic role of PLR in CRC and reported that elevated PLR was significantly associated with poor prognosis [30, 31]. Our study was based on the northern Chinese population and had some new findings. First, we used a new statistical method to determine the optimal cut-off of PLR. Furthermore, we evaluated the additional prognostic value of PLR in the current TNM staging system. We found that that TNM+PLR had a better prognostic capacity than TNM staging alone. Therefore, PLR may serve as an additional parameter in the current TNM staging system and may increase the accuracy for predicting the prognosis of CRC patients.

This study has several limitations. First, the data of the cohort patients were analyzed retrospectively. Second, because of the long data collection period in this retrospective cohort analysis, advances in surgery and chemotherapy during this period may affect clinical outcomes. Third, owing to the lack of relevant data, the relationship between PLR and adjuvant chemotherapy was not explored.

In conclusion, our results indicated that elevated PLR was a poor prognostic indicator in CRC patients, and PLR might serve as a complement to the current TNM staging system for predicting CRC.

## MATERIALS AND METHODS

### Study patients

Data on consecutive patients who underwent curative resection of CRC between January 2000 and May 2014 at the Department of Surgical Oncology at the First Hospital of China Medical University (CMU-SO) were collected retrospectively. Patients who met the following criteria were selected: (1) CRC was diagnosed by histopathology; (2) patients did not receive neoadjuvant therapy or anti-inflammatory treatment before surgery; and (3) laboratory tests were conducted before surgery. Patients who underwent surgery in emergency circumstances, including obstruction and perforation, were excluded. Patients with liver cirrhosis, portal vein hypertension, past history of heart disease, and long-term use of anticoagulant drugs were not included in this study. After this selection, 1845 patients were included in our study. Follow-up was completed for all patients until October 2015. The median follow-up period was 50 months. Preoperative laboratory data and relevant clinicopathological variables, including tumor size, tumor location, tumor stage, and differentiation grade, were recorded. All cases were restaged according tothe seventh edition of the AJCC/UICC TNM classification system.

### Statistical analysis

The associations between categorical variables were analyzed using the chi-square test. The association between PLR and prognosis, including OS and CSS, was determined using the Kaplan-Meier method with the log-rank test. Multivariate analysis was conducted using the Cox proportional hazards model. The predictive capacity of the different categories was evaluated by measuring discrimination, which refers to the ability to distinguish between high-risk and low-risk patients. We used c-index [32, 33] to quantify discrimination. A model with perfect predictive capacity would have a c-index of 1.00. A greater c-index indicates a better model for predicting the outcome.

In this study, the novel category TNM+PLR, which combined TNM staging with PLR, was based on the hazard ratio (HR) calculated by the Cox proportional hazards model. The formula of the Cox proportional hazards model is H(t)/H0(t) = exp (β_1_x_1_+β_2_x_2_+β_3_x_3_+…+β_k_x_k_), where x_1_…x_k_ are a collection of predictor variables, TNM and PLR in this study, β_1_…β_k_ are regression coefficients determined by a least squares approach, and the H(t)/H0(t) is the HR. Moreover, we grouped the calculated HR into four risk levels and formed the novel category TNM+PLR using the method of c-index.

Statistical evaluation was performed using SPSS software version 22.0 (SPSS, Chicago, IL, USA) and STATA software (version 12.0, Stata Corporation, College Station, TX, USA). All statistical tests were two-sided, and a *P*-value of less than 0.05 was considered statistically significant.

## SUPPLEMENTARY MATERIALS TABLE



## Abbreviations

CI: confidence interval
c-index: Harrell’s concordance index
CMU-SO: the Department of Surgical Oncology at the First Hospital of China Medical University
CRC: colorectal cancer
CSS: cancer-specific survival
HR: hazard ratio
OS: overall survival
PLR: platelet to lymphocyte ratio
