# Supplementary material for: Prognostic evaluation of platelet to lymphocyte ratio in patients with colorectal cancer
Source: Oncotarget. 2017 Sep 21;8(49):86287–95. doi: 10.18632/oncotarget.21141 (PMC5689685; doi:10.18632/oncotarget.21141)
Supplement: Supplementary file 1 [file oncotarget-08-86287-s001.pdf]

Prognostic evaluation of platelet to lymphocyte ratio in patients with colorectal cancer

SUPPLEMENTARY MATERIALS

Supplementary Table 1: The five greatest c-index values of different cut-off values for PLR for predicting prognosis in CRC patients from CMU-SO

Survival	Cut-off	c-index	N	Survival	Cut-off	c-index	N
**OS**	130	0.5494	1018/827	**CSS**	130	0.5553	1018/827
	132	0.5481	1048/797		129	0.5543	1006/839
	129	0.5481	1006/839		132	0.5539	1048/797
	131	0.5470	1036/809		131	0.5530	1036/809
	133	0.5462	1067/778		127	0.5525	978/867

CSS: cancer-specific survival; CMU-SO: Department of Surgical Oncology at The First Hospital of China Medical University; N: number of patients for each group; OS: overall survival; PLR: platelet to lymphocyte ratio.
